# Nationwide Assessment of Polycyclic Aromatic Hydrocarbons (PAHs) in Indoor Dust Across China: Pollution Characteristics, Sources, and Particle Size Distribution

**DOI:** 10.3390/toxics13100821

**Published:** 2025-09-26

**Authors:** Mei-Hua Tian, Wen-Long Li, Liang Wang, Ting Cai, Shuang Du, Xin-Hong Wang, Chun-Yan Huo

**Affiliations:** 1 College of the Environment and Ecology, Xiamen University, Xiamen 361102, China; 2 Third Institute of Oceanography, Ministry of Natural Resources, Xiamen 361005, China; 3 CNPC Research Institute of Safety and Environmental Technology, Beijing 102206, China; 4 Institute of NBC Defense, Beijing 102205, China

**Keywords:** anthropogenic contaminants, indoor dust, particle size distribution

## Abstract

Polycyclic aromatic hydrocarbons (PAHs) in indoor dust pose significant health risks due to their persistence and carcinogenicity. This study comprehensively evaluates PAH concentrations, spatial distribution, sources and particle size distribution in indoor dust collected across 26 Chinese provinces. Each dust sample was fractionated into six fractions: F1 (1000–2000 μm), F2 (500–1000 μm), F3 (250–500 μm), F4 (125–250 μm), F5 (63–125 μm), F6 (<63 μm). The total concentration of the 17 PAHs (∑_17_PAHs) ranged from 0.63 to 247 μg·g^−1^, with a median value of 4.3 μg·g^−1^. High ∑_17_PAH concentrations were found in North China. PAHs with three rings and four rings were the most prevalent PAHs, accounting for 80.8% of ∑_17_PAHs. PAH concentration is negatively correlated with dust particle size, and there are certain differences in the particle size distribution patterns of different types of dust samples. The diagnostic ratios and principal component analysis (PCA) indicated that indoor dust mainly originated from fuel combustion and traffic emissions.

## 1. Introduction

Polycyclic aromatic hydrocarbons (PAHs) are a large class of organic compounds composed of carbon and hydrogen atoms, typically containing two to seven aromatic rings. PAHs exhibit characteristics such as long-term persistence and bioaccumulation, and can persist in the environment [1]. PAHs have been widely detected in various environmental media, including the atmosphere [2], soil [3], rivers [4], marine environment [5], food [6], and dust [7]. Exposure to PAHs can pose many adverse health effects on the human body, such as increasing the risk of cancer, causing birth defects in infants, and damaging the immune and nervous systems [8,9].

Indoor PAHs originate from diverse sources, such as cooking, tobacco smoking, the use of decorative materials, and everyday consumer products [10,11]. In addition, polluted air containing PAHs can be generated by outdoor fossil fuel combustion, traffic, and industrial emissions, which will enter the indoor environment through window ventilation and the use of air conditioners [12,13]. Numerous studies have reported PAH contamination in indoor settings. For example, the total concentrations of PAHs detected in household dust from eight cities across China ranged from 0.040 to 360 μg·g^−1^, markedly exceeding those reported for most countries worldwide [14]. The average value of ∑PAHs collected from Zhengzhou University campuses was 2.4 μg∙g^−1^, indicating potential carcinogenic risks to students [15]. However, comprehensive information on nationwide PAH contamination in indoor dust is still limited [16].

Particle size is a key physical parameter influencing contaminant distribution in indoor dust. Fine particles possess larger specific surface areas, facilitating both the adsorption of pollutants and their subsequent uptake [17]. Moreover, dust particle size is a crucial factor in evaluating human exposure, as smaller particles are more easily ingested and adhere to the skin [18,19]. He et al. found that organophosphate flame retardants in household and building material market dust preferentially accumulated in particles <150 μm [20]. Liu et al. reported that PAHs in dust from five microenvironments in Nanjing (offices, laboratories, classrooms, lobbies, and supermarkets) were consistently enriched in fine fractions [21]. However, research on the size distribution of indoor dust particles collected nationwide remains limited, highlighting the need for further investigation into how pollutants are distributed across different particle sizes on a large scale.

Although numerous investigations have reported PAH contamination and carcinogenic risk in indoor dust, most have focused on one or a few regions. Several studies indicate that PAH concentrations in Chinese indoor dust are among the highest worldwide and may be influenced by geographical location [22,23,24]. For instance, the concentration of PAHs in the indoor dust of 23 cities in China ranged from 1.0 μg∙g^−1^ to 470 μg∙g^−1^ with a mean value of 31 μg∙g^−1^, and a correlation was found between the PAH concentration in indoor dust and geographical location [25]. Liu et al. collected household dust samples from 27 provinces and 1 municipality in China, and found that the ∑_14_PAH concentrations ranged from 3.7 to 61,000 ng∙g^−1^, with the highest levels in Northeast and Southwest China [26]. Many other existing studies focused on individual or limited regional scales, leaving a knowledge gap regarding the pollution characteristics of PAHs in indoor dust across China [27,28].

To address these gaps, this study collected and analyzed indoor dust samples from 26 provinces, municipalities, and autonomous regions in China. The primary objectives were (a) to analyze the concentrations, compositional profiles, and spatial distribution of PAHs in Chinese indoor environments; (b) to examine the particle size distribution patterns of PAHs in indoor dust; (c) to identify the potential sources of indoor PAHs in China. By addressing these objectives, this study provides comprehensive insights into the nationwide status of PAH contamination in indoor dust, offering valuable data for pollution control strategies. This study also provides historical data that, when compared with other datasets, helps to establish temporal trends in PAH contamination in indoor environments across China.

## 2. Materials and Methods

### 2.1. Chemicals and Reagents

The full name and abbreviation of the 17 PAH standards are shown in Table 1. These standards and internal standards (naphthalene-D8, fluorene-D10, pyrene-D10, and perylene-D12) were purchased from Sigma Aldrich (St. Louis, MO, USA). All solvents (acetone, n-hexane, dichloromethane, and iso-octane) used during the entire experiment were chromatographically pure and purchased from J. T. Baker (Waltham, MA, USA).

### 2.2. Indoor Dust Collection

A nationwide sampling campaign in July–August 2016 collected single indoor dust samples from 73 residential sites across China, including 41 urban and 32 rural samples, all located away from highways and contaminated environments. Among them, 48 samples can be considered as home samples (including residential apartments and dormitories), and 25 as public samples (including offices, classrooms, commercial stores, and laboratories), based on their locations. Therefore, all the samples were divided into 4 types: rural private homes, rural public houses, urban private homes, and urban public houses. The spatial distributions of indoor dust sampling sites are shown in Figure S1 in the Supporting Information (SI).

Dust samples were collected by trained volunteers using small brushes. Samples from different locations at each site were pooled and homogenized to form a representative sample. After gross impurities and debris such as small pellets, short and long fibres, hairs, small stones, and glass pieces were discarded, the dust samples were individually fractionated into 6 categories depending on particle size measured through different stainless steel sieves [F1 (1000−2000 μm), F2 (500−1000 μm), F3 (250−500 μm), F4 (125−250 μm), F5 (63−125 μm), and F6 (<63 μm)], and then stored using clean aluminum foil in a refrigerator at −20 °C until treatment. The brushes, stainless steel sieves, and aluminum foil were pre-cleaned with acetone and hexane before use to avoid cross-contamination.

### 2.3. Pretreatment and Analysis

Ultrasonic extraction was used to extract PAHs from the indoor dust samples. The detailed procedures are as follows: Each dust sample was placed into a 10 mL glass centrifuge tube spiked with surrogates (Naphthalene-D_8_, fluorene-D_10_, pyrene-D_10_, and perylene-D_12_) and mixed with a mixture of hexane/acetone (4/1, *v/v*). Then, samples were ultrasonically extracted for 30 min before shaking extraction for 30 min, and centrifuged at 4000 rpm for 5 min. The supernatant solvent was transferred to a new glass tube. All extractions were repeated three times, and the extraction solvents were mixed. After the extracts were purified using a silica gel column, the pooled extracts were concentrated and solvent-exchanged with isooctane to 1 mL under a gentle stream of nitrogen, and stored at −20 °C before instrumental analysis. All glassware used during sample pretreatment was kept at 400 °C overnight before use.

Quantitative analysis of PAHs was performed using gas chromatography (Agilent Technologies 6890 N, Santa Clara, CA, USA) coupled with mass spectrometry (Agilent Technologies 5975B, Santa Clara, CA, USA) in electron capture negative ionization mode, and separation was conducted using an HP5-MS capillary column (30 m × 0.25 mm i.d., 0.25 μm film thickness, J&W Scientific, Santa Clara, CA, USA) with helium as the carrier gas at a flow rate of 1.5 mL/min. Initially, the oven temperature was programmed from 80 °C (held for 3 min), then raised to 300 °C at 10 °C/min, and held for 5 min. The sample extract (each 2 μL) was injected in a pulsed splitless mode at an injector temperature of 265 °C. The temperatures of the quadrupole, ion source, and interface were maintained at 150 °C, 230 °C, and 250 °C, respectively. The selected ion monitoring (SIM) mode was used for MS. The identities of the PAHs were confirmed based on the selected ions and comparison of the retention time between samples and the PAH standard solution.

### 2.4. QA/QC

To ensure quality control and evaluate method accuracy, one procedural blank sample and one spike blank sample were analyzed in parallel with each batch of samples (10 dust samples for one batch) to check for contamination during sampling, transport, treatment, and instrumental analysis. Pre-cleaned anhydrous sodium sulfate powder was prepared as a surrogate for the dust sample. All clean-up and extraction steps were performed under a fume hood without light to avoid photodegradation. All the glassware was thoroughly rinsed with acetone and n-hexane before use. The recoveries of target PAH congeners in the spiked samples ranged from 76% to 114%, which indicated the good performance of the treatment method. The concentrations of most PAHs in the procedural blank samples were detected at levels less than 5% of PAHs in real dust samples, except for acenaphthene (15%) and fluorene (9%). The reported concentrations of PAHs in real samples were not blank-corrected in this study. The recoveries of surrogate standards in dust samples ranged from 72% to 109% for naphthalene-D8, 72% to 122% for fluorene-D10, 83% to 128% for pyrene-D10, and 76% to 118% for perylene-D12. The regression coefficients (R) of all instrumental calibration curves were ≥0.99. Statistical analyses were performed using SPSS Statistics 27, with statistical significance set at *p* < 0.05.

## 3. Results

### 3.1. Overview of PAH Concentrations in Indoor Dust

#### 3.1.1. Concentrations of PAHs

Table 1 displays the descriptive statistics of PAH concentrations analyzed in indoor dust samples. The concentrations of ∑_17_PAHs in indoor dust were 0.63–247 μg∙g^−1^, with a mean and median of 9.6 μg∙g^−1^ and 4.3 μg∙g^−1^, respectively. Phe was the dominant PAH with a median concentration of 0.81 μg∙g^−1^, followed by Flt, Chr, BeP, and Pyr, median concentrations of 0.73 μg∙g^−1^, 0.47 μg∙g^−1^, 0.46 μg∙g^−1^, and 0.46 μg∙g^−1^, respectively.

**Table 1:** Minimum (Min), maximum (Max), mean, standard deviation (Std), median, 5th percentile (P5th), and 95th percentile (P95th) concentrations (μg∙g^−1^) of PAHs in indoor dust collected from China.

PAHs	Abbreviation	Min	Max	Mean	Std	Median	P5th	P95th
Acenaphthylene	Acy	0.0033	1.3	0.076	0.17	0.035	0.0090	0.24
Acenaphthene	Ace	0.0043	0.87	0.059	0.11	0.031	0.012	0.14
Fluorene	Flu	0.022	3.1	0.23	0.39	0.13	0.049	0.48
Phenanthrene	Phe	0.089	58	2.1	6.91	0.81	0.21	4.19
Anthracene	Ant	0.0048	3.1	0.13	0.38	0.047	0.011	0.35
Fluoranthene	Flt	0.096	89	2.4	11	0.73	0.15	3.3
Pyrene	Pyr	0.075	63	1.7	7.5	0.46	0.10	2.2
Benzo(a)anthracene	BaA	0.011	5.0	0.26	0.61	0.13	0.031	0.58
Chrysene	Chr	0.070	14	0.80	1.7	0.47	0.10	2.2
Benzo(b)Fluoranthene	BbF	0.030	3.3	0.40	0.45	0.26	0.052	0.86
Benzo(k)Fluoranthene	BkF	0.016	1.7	0.23	0.24	0.17	0.031	0.61
Benzo(e)Pyrene	BeP	0.053	2.8	0.59	0.52	0.46	0.081	1.6
Benzo(a)Pyrene	BaP	0.0078	0.75	0.15	0.15	0.10	0.023	0.47
Perylene	Per	0.0038	0.31	0.05	0.057	0.034	0.0060	0.17
Indeno(123-c,d)Pyrene	IcdP	0.012	0.79	0.18	0.16	0.14	0.024	0.45
Dibenzo(ah)anthracene	DahA	0.0011	0.27	0.06	0.055	0.035	0.0060	0.15
Benzo(g,h,i)Perylene	BghiP	0.012	0.88	0.21	0.18	0.17	0.029	0.50
	∑_17_PAHs	0.63	247	9.6	29	4.3	1.1	17

#### 3.1.2. Housing Types

The concentrations of PAHs in indoor dust in urban and rural areas ranged from 1.1 to 18 μg∙g^−1^ and 0.63 to 247 μg∙g^−1^. The median concentration of PAHs in rural indoor dust (4.0 μg∙g^−1^) was lower than that in urban areas (4.7 μg∙g^−1^). There was no significant difference in the total concentration of PAHs in indoor dust between urban and rural areas (*p* > 0.05) (Figure S2).

As shown in Figure S2, the median concentration of PAHs in public dust (5.9 μg∙g^−1^) was significantly higher than that in household buildings (3.6 μg∙g^−1^) (*p* < 0.05). In addition, some high concentrations of PAHs were found in public buildings (up to 247 μg∙g^−1^). For different types of public places, the median PAH concentrations in stores (12 μg·g^−1^) and other public places (9.1 μg·g^−1^) were significantly higher than those in schools (5.8 μg·g^−1^) (*p* < 0.05), with offices showing the lowest level (3.8 μg·g^−1^).

### 3.2. Spatial Distribution

**Figure 1:**
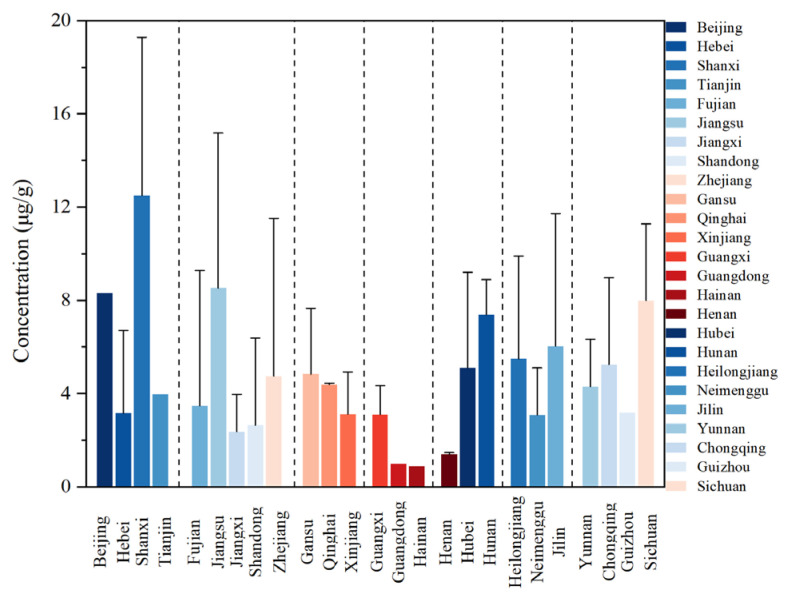
Distribution characteristics of ∑_17_PAHs in dust from Chinese provinces (median ± standard deviation).

The spatial distribution of ∑_17_PAHs at each province is shown in Figure 1. The median concentration of ∑_17_PAHs was found to be the highest in Shanxi (17 μg∙g^−1^), followed by Jiangsu province (12 μg∙g^−1^). In contrast, the concentrations of ∑_17_PAHs in Henan (1.4 μg∙g^−1^), Guangdong (0.98 μg∙g^−1^), and Hainan (0.91 μg∙g^−1^) are relatively lower. Geographically, high PAH levels were predominantly located in North China (63 μg∙g^−1^), while lower concentrations clustered in South China (1.7 μg∙g^−1^) (Figure 1).

### 3.3. Compositional Characteristics

**Figure 2:**
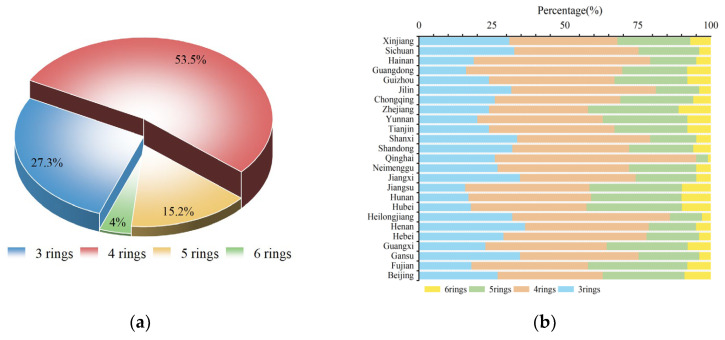
National distribution of PAHs by ring number in indoor dust (**a**). Provincial distribution of PAH ring numbers in indoor dust across China (**b**).

To obtain insight into the compositional patterns of PAHs in indoor dust in China, the relative contents of compounds with different ring numbers to the ∑_17_PAH contents were calculated, and the results are shown in Figure 2a. In this study, high-molecular-weight PAHs (HMW, four–six-ring PAHs) dominated the ∑_17_PAHs in Chinese indoor dust, accounting for 72.7% of the total PAHs. Among these, four-ring PAHs showed the highest proportion (53.5%), followed by three-ring PAHs (27.3%).

### 3.4. Particle Size Distribution Patterns

**Figure 3:**
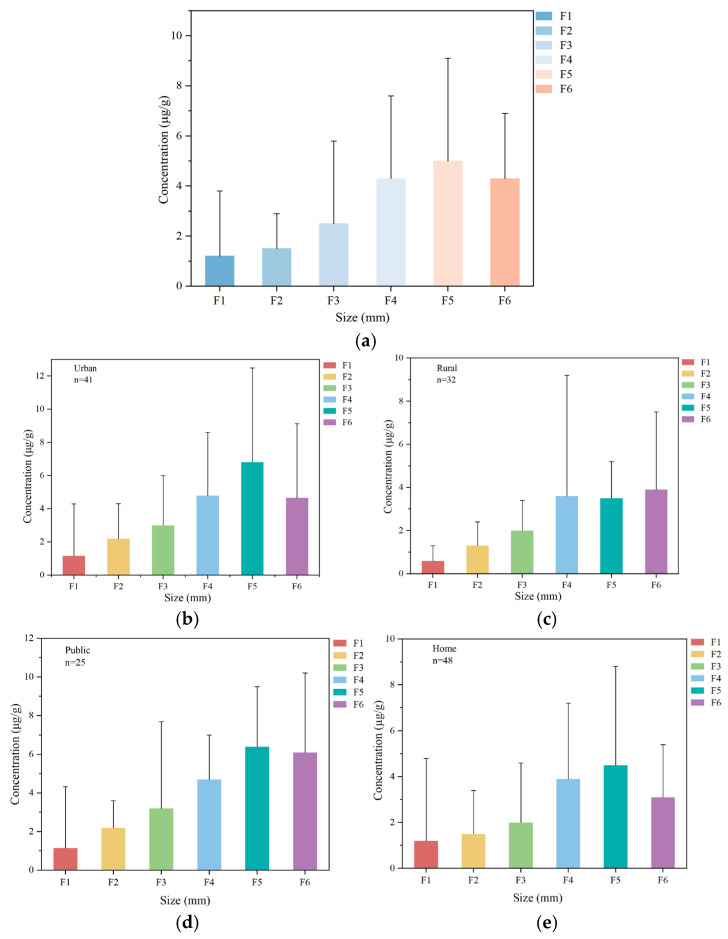
Concentrations of PAHs in indoor dust across different particle sizes (median ± standard deviation): (**a**) ∑_17_PAHs in indoor dust; (**b**) urban indoor dust; (**c**) rural indoor dust; (**d**) public indoor dust; and (**e**) home indoor dust.

The size-dependent distributions of PAHs in all indoor dust samples are shown in Figure 3a. PAHs were preferentially concentrated in particles <250 μm, with concentrations showing a steady increase as particle size decreased and a significant peak at F5 (63−125 μm). There are significant differences in PAH concentrations among dust with different particle sizes (Kruskal–Wallis H, *p* < 0.05).

Regional size distribution patterns are shown in Figure 3b–e and reveal similar features. In rural dust, PAHs were significantly enriched in F4–F6, with levels markedly higher than those in other fractions. Urban dust peaked in F5, where concentrations were approximately three times those of the coarsest fraction (F1). Across building types, PAH levels increased steadily with decreasing particle size, peaking at F5. Household dust had the highest PAH concentrations in F4–F5, whereas F5–F6 of public dust showed higher PAH values than other fractions.

The size distribution patterns of PAHs grouped by ring number were generally similar (Figure S3a–b). All PAHs were enriched in finer fractions (F4–F6), accounting for 60–82% of the total load. The concentration of three-ring PAHs peaked in F4 (24%), followed by F5 (23%), collectively accounting for approximately 50% of their total load. Four-ring PAHs were concentrated in the range F4–F6 (75%), with the peak occurring at F5. The PAHs with six and five aromatic rings exhibited continuous concentration increases with decreasing particle size, peaking in F6 and contributing over 30% of their respective totals.

### 3.5. Sources of PAHs

PAHs from different sources exhibit distinct molecular compositions, and thus the diagnostic ratio is widely used to identify the probable origins of PAHs in different environmental media [29,30]. In this study, the ratios of BaA/(BaA+CHR), IcdP/(IcdP+BghiP), and Flu/(Flu+Pyr) were employed to identify the sources of PAHs in indoor dust. When the BaA/(BaA+Chr) and IcdP/(IcdP+BghiP) ratios are <0.2, this represents petroleum sources; ratios in the range 0.2 to 0.35 and 0.2 to 0.5, respectively, indicate that coal and biomass combustion are the primary sources of PAHs in indoor dust; and ratios > 0.35 and 0.5 point to coal and biomass as the primary sources [31,32,33]. As for Flu/(Flu+Pyr), ratios < 0.4 indicate petroleum spills; those in the range of 0.4 to 0.5 suggest fossil fuel combustion, and those > 0.5 imply the low-temperature combustion of coal and biomass [34].

**Figure 4:**
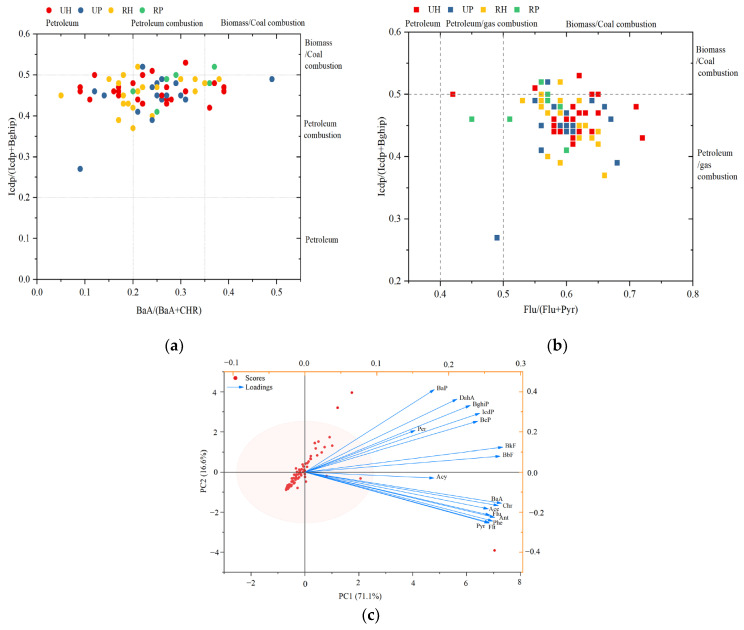
Characteristic ratio (BaA/(BaA+CHR), IcdP/(IcdP+BghiP) analysis for PAH source identification (**a**). Characteristic ratio (BaA/(BaA+CHR), Flu/(Flu+Pyr) analysis for PAH source identification (**b**). Source apportionment of indoor PAHs by principal component analysis (**c**). Notes: UH, urban home; UP, urban public; RH, rural home; and RP, rural public.

In this study, the diagnostic ratios in the dust from China are shown in Figure 4a,b. The BaA/(BaA+Chr) ratios were <0.35, the IcdP/(IcdP+BghiP) ratios ranged from 0.2 to 0.5, and the Flu/(Flu+Pyr) ratios were >0.5 (Figure 4a,b). The sources of PAHs in indoor dust from both rural and urban areas, as well as from domestic and public settings, are similar, primarily originating from the combustion of petroleum, coal, and biomass, with a minor contribution from petroleum leakage.

To further identify the source of PAHs in indoor dust from China, principal component analysis (PCA) was carried out based on PAH concentrations in indoor dust (Figure 4c). The two principal components can be used to conduct the source apportionment of PAHs in indoor dust, as they summarize 87.7% of the original information. PC1 accounted for 71.1% of the total variance of PAHs, among which are BkF, BbF, BaA, Chr, Ant, Pyr, and Phe. BkF and BbF showed greater loading values. BkF and BbF are markers of fossil fuel combustion [35,36]; BaA, Chr, and Phe mainly originate from coal and natural gas [37,38,39]; and Phe and Pyr are tracers of diesel vehicle emissions [25,40]. Thus, PCA1 represents combustion and traffic emission sources. PCA2 explained 16.6% of the total variance with high loadings of BaP, DahA, and BghiP, which are associated with vehicle exhaust emissions [41,42]. Therefore, PCA2 represents traffic-related sources.

## 4. Discussion

### 4.1. Pollution of PAHs in Indoor Dust

A temporal comparison of PAH levels in Chinese indoor dust over the past fifteen years is illustrated in Figure S4 and Table S1. Overall, the concentrations of PAHs in indoor dust in China show a downward trend. The average concentrations of PAHs ranged from 0.61 to 54 μg∙g^−1^. The highest average concentration of PAHs in indoor dust was reported in 2018 (54 μg∙g^−1^) [14], followed by the studies reported in 2012 and 2010, with PAH concentrations of 35 μg∙g^−1^ [43] and 31 μg∙g^−1^ [25], respectively. The decrease in PAHs during this period was likely due to heightened environmental awareness and improved pollution control measures, which have collectively reduced the emissions of PAHs into both indoor and outdoor environments. Nevertheless, comparisons among studies should be interpreted cautiously because total PAH concentrations are influenced by dust particle size fractions, sampling locations, and other factors [15]. It should be noted that this study is primarily spatial in scope, and the samples were collected over multiple years during various field campaigns. While this approach enables broad geographical assessment, it does not capture high-resolution temporal variations (e.g., seasonal or monthly trends) at individual sites. Future long-term monitoring studies are recommended to better characterize the temporal dynamics of indoor dust PAHs and refine exposure assessments.

Relative to other countries, the concentrations of ∑_17_PAHs in this study were much higher than most studies in other countries, such as Greece (1.9 μg∙g^−1^, sampling year: 2018) [44], Kuwait (1.1 μg∙g^−1^, sampling year: 2018) [45], Serbia (1.8 μg∙g^−1^, sampling year: 2021) [46], Iran (0.16 μg∙g^−1^, sampling year: 2020) [30], Nigeria (2.6 μg∙g^−1^, sampling year: 2019) [47], and Thailand (3.9 μg∙g^−1^, sampling year: 2023) [48]. The notably higher PAH burdens in Chinese indoor dust are presumably attributable to the coal-dominated energy structure and the rapid increase in vehicular emissions [14].

For the median PAH concentrations in different types of indoor dust, the concentrations in cities are slightly higher than those in rural areas. Qi et al. also reported that the concentration of PAHs in urban indoor dust (37 μg∙g^−1^) was higher than that in rural areas (16 μg∙g^−1^) [25]. These findings align with previous reports on PAH concentrations in household versus public building dust across China. The elevated PAH levels in some public buildings may be attributed to traffic-related outdoor sources [25].

### 4.2. Spatial Distribution Characteristics

Existing studies indicate that PAHs primarily originate from fuel combustion [49]. The sampling sites with high ∑_17_PAHs in household dusts in these areas are located in rural households, where biomass (e.g., firewood) and coal are commonly used as cooking fuels, leading to higher PAH accumulation in indoor environments compared to other sampling sites [26]. Previous investigations have likewise documented higher PAH concentrations in Shanxi province compared to other provinces, likely resulting from abundant coal resources and their subsequent utilization for residential cooking and heating in this region [49]. The different concentrations of PAH in indoor dust in different regions of China demonstrate significant spatial variability influenced by geographical factors.

### 4.3. Compositional Characteristics

The abundance order of PAHs with different numbers of rings was as follows: four rings > three rings > five rings > six rings. The compositional patterns of PAHs in indoor dust from different provinces were similar (Figure 2b), indicating a rather uniform PAH profile in indoor dust across China. The existing literature confirms that HMW PAHs are the major constituents of PAHs in indoor dust [50,51,52]. High-molecular-weight PAHs were also found to be dominant in the indoor dust collected from Serbian households, with four-ring (40–53%) and three-ring PAHs (29–40%) accounting for the highest proportion of the total PAHs [46]. PAHs in various types of indoor dust from Changchun, China, also showed a predominance of HMW PAHs [27]. A similar allocation of PAHs was observed in indoor dust collected from school campuses in Henan, with HMW PAHs (71.8%) being more abundant than LMW PAHs (27.8%) [24]. The elevated detection of HMW PAHs in indoor dust may be attributed to their greater stability and tendency to persist in the particulate phase by adsorbing onto dust particles. In contrast, LMW PAHs are more volatile and primarily exist in the gas phase [16,53].

### 4.4. Particle Size Distribution Pattern

The particle size distribution pattern of PAHs is mainly concentrated in fine-sized dust. This pattern is similar to that reported for indoor environments in Beijing (hotels, offices, kindergartens, and dormitories) [54], and for road dust and indoor parking lot dust in Guangzhou [55]. These findings suggest that PAHs are more likely to adhere to finer dust particles, possibly due to their larger specific surface area, which facilitates the accumulation of pollutants [56]. The pattern of indoor dust from rural and urban areas was similar to the overall dust distributions in all sampling sites. The differing particle size distribution patterns of PAHs among indoor dust types may be attributed to variations in PAH sources and dust composition [54]. Overall, PAHs with different numbers of rings also show a similar distribution pattern. PAHs with higher ring numbers showed a greater tendency to accumulate on finer particles.

### 4.5. Source Apportionment of PAHs

The diagnostic ratios of BaA/(BaA+Chr), IcdP/(IcdP+BghiP), and Flu/(Flu+Pyr) identified traffic emissions and fuel combustion as the predominant sources of PAHs in Chinese indoor dust. Previous studies covering ten provinces in China likewise identified indoor fuel combustion and outdoor traffic emissions as the dominant sources of PAHs in indoor dust [57]. In Guangzhou, China, the sources of PAHs in indoor dust were also found to be mainly from fuel combustion emissions [22]. Liu et al. further reported that household cooking and heating activities are the primary contributors to PAHs in household dust across China [26]. Source-oriented control is necessary to reduce the content of PAHs in residential dust [14]. The main sources of PAHs in indoor dust in China can be preliminarily attributed to fuel combustion emissions, including household energy use and traffic-related activities. Therefore, promoting the use of clean energy and new energy vehicles may help reduce the emission of polycyclic aromatic hydrocarbons and improve indoor air quality.

### 4.6. Limitations

The main limitations of this study are as follows: First, the number of sampling sites within each province was limited and unevenly distributed, which may restrict the spatial representativeness of PAH pollution in indoor dust at the provincial scale. Second, metadata associated with sampling locations (e.g., building age, ventilation patterns, floor type, and household cleaning frequency) were not systematically collected, limiting our ability to conduct a comprehensive analysis of factors influencing PAH concentrations. Consequently, spatial distribution and source attribution remain preliminary and should be interpreted with caution.

## 5. Conclusions

This study probed into the concentrations of PAHs in indoor dust from China. The total concentrations of 17 PAHs (∑_17_PAHs) analyzed in indoor dust samples of China ranged from 0.63 to 247 μg∙g^−1^, with a median value of 4.3 μg∙g^−1^. Phe, Flt, Chr, BeP, and Pyr were the dominant PAHs in all indoor dusts. High ∑_17_PAHs in indoor dusts were mainly distributed in North China. The HMW PAHs found in China’s indoor dust greatly contributed to the total PAHs, comprising up to 72.7% of the ∑_17_PAH level. A comparison with previous literature indicates that the concentration of ∑_17_PAHs in China shows a downward trend. However, the concentrations of ∑_17_PAHs in this study were much higher than those reported in most studies in other countries. PAHs in indoor dust generally tended to concentrate in finer particle fractions, and different sampling regions showed different particle size distribution patterns. The diagnostic ratios of BaA/(BaA+Chr), IcdP/(IcdP+BghiP), and Flu/(Flu+Pyr), together with principal component analysis (PCA), identified traffic emissions and fuel combustion as the predominant sources of PAHs in Chinese indoor dust.

## Data Availability

Data will be made available on request.

## Supplementary Materials

The following supporting information can be downloaded at: https://www.mdpi.com/article/10.3390/toxics13100821/s1, Figure S1: Distribution map of national indoor dust sampling sites; Figure S2: The total concentrations of PAHs in indoor dust from different types of areas (Mann–Whitney U test); Figure S3: Distribution of PAHs in dust of different particle sizes across different ring numbers: (**a**) 3 rings; (**b**) 4 rings; (**c**) 5 rings; and (**d**) 6 rings; Figure S4: The total concentrations of PAHs in indoor dust from China during the period of 2010–2020; Table S1: Mass fraction ranges of PAHs in indoor dust in China (μg∙g^−1^). Refs. [14,22,23,24,25,26,28,43,57,58,59,60,61,62] are cited in the Supplementary Materials.
