# Supplementary material for: Nationwide Assessment of Polycyclic Aromatic Hydrocarbons (PAHs) in Indoor Dust Across China: Pollution Characteristics, Sources, and Particle Size Distribution
Source: Toxics. 2025 Sep 26;13(10):821. doi: 10.3390/toxics13100821 (PMC12568245; doi:10.3390/toxics13100821)
Supplement: Supplementary file 1 [file toxics-13-00821-s001.zip › toxics-3836176-supplementary.pdf]

Supplementary Materials

Nationwide Assessment of Polycyclic Aromatic Hydrocarbons (PAHs) in Indoor Dust across China: Pollution Characteristics, Sources, and Particle Size Distribution

Mei-Hua Tian a, Wen-Long Li a, Liang Wang b, Ting Cai c, Shuang Du d, Xin-Hong Wang a,*, Chun-Yan Huoa,*

* Corresponding authors. Chun-Yan Huo, e-mail address: huochunyan1119@163.com. Xin-Hong Wang, e-mail address: xhwang@xmu.edu.cn.

[1] College of the Environment and Ecology, Xiamen University, Xiamen 361102, China

[2] Third Institute of Oceanography, Ministry of Natural Resources, Xiamen 361005, China

[3] CNPC Research Institute of Safety and Environmental Technology, Beijing 102206

[4] Institute of NBC Defense, Beijing 102205, China

Table of Contents

Nationwide Assessment of Polycyclic Aromatic Hydrocarbons (PAHs) in Indoor Dust across China: Pollution Characteristics, Sources, and Particle size distribution 1

S1. Supporting Tables 3

Table S1. Mass fraction ranges of PAHs in indoor dust in China (μ g·g^{-1}). 3

S2. Supporting figures 4

Figure S1. Distribution map of national indoor dust sampling sites. 4

Figure S2. The total concentrations of PAHs in indoor dust from different types of areas. (Mann-Whitney U test) 5

Figure S3. Distribution of PAHs in dust of different particle sizes across different ring numbers: (a) 3 rings; (b) 4 rings; (c) 5 rings; (d) 6 rings. The bars represent the concentration of PAHs (μg·g^{-1}), and the dotted curves represent the percentage contribution of each ring-number group to the total $\sum_{17}$PAHs. 6

Figure S4. The total concentrations of PAHs in indoor dust from China during the period of 2010 – 2020. Detailed data can be found in Table S1. 7

S4. References 8

S1. Supporting Tables

Table S1. Mass fraction ranges of PAHs in indoor dust in China (μg·g⁻¹).

Measuring Place	Sampling Year	No. of Measured PAHs	ΣPAH	Mean	References
23 cities	2010	16	1.0–470	31	[1]
Nanjing	2011	16	1.2–280	16	[2]
Guizhou	2012	18	2.2–14	6.8	[3]
Xinxiang	2012	16	1.5–22	8.4	[4]
Qingyang	2012	16	8.5–121	35	[5]
Guangzhou	2017	16	1.8–14	6.1	[6]
Northern China	2017	16	/	3.8	[7]
Dalian	2018	16	1.6–26	6	[8]
8 cities	2018	16	0.040–356	54	[9]
Wuhan	2019	16	2.61–10.6	5.2	[10]
Xinxiang	2020	16	/	2.1	[11]
10 provinces	2020	16	0.61–10	3.5	[12]
28 provinces	2021	14	0.0037 −61	4.1	[13]
South China	2021	15	0.103–1.055	0.61	[14]

S2. Supporting figures

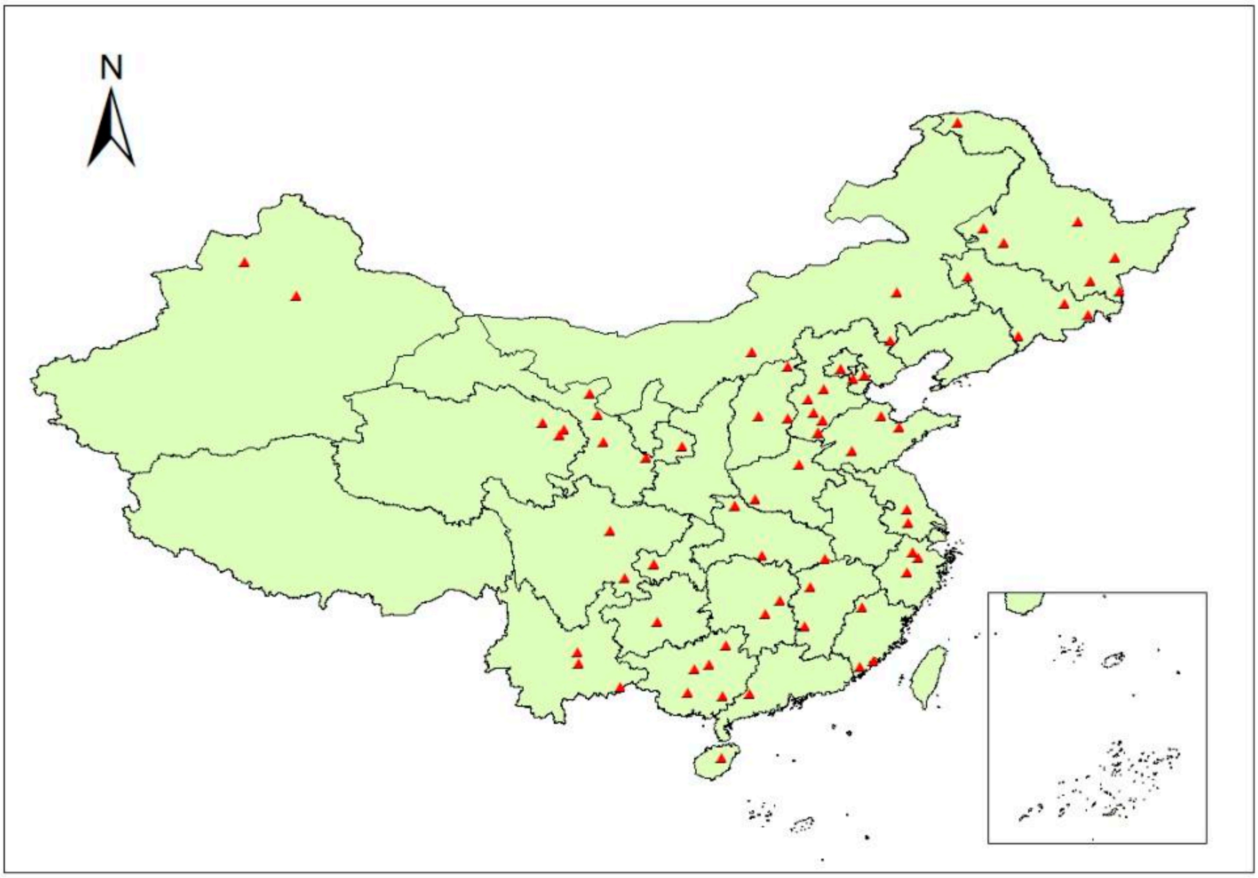

Figure S1. Distribution map of national indoor dust sampling sites.

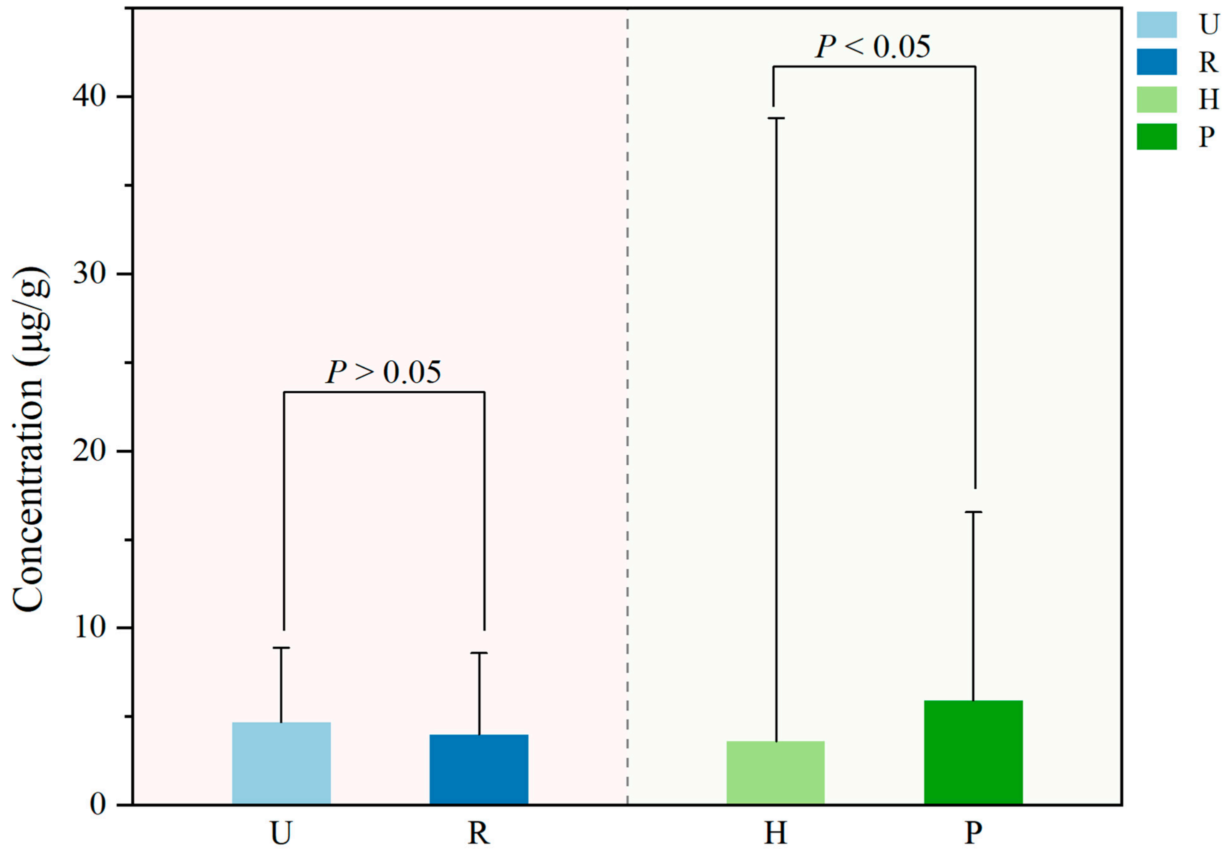

Figure S2. The total concentrations of PAHs in indoor dust from different types of areas. (Mann-Whitney U test)

Note: U, Urban; P, Public; H, Home; P, Public.

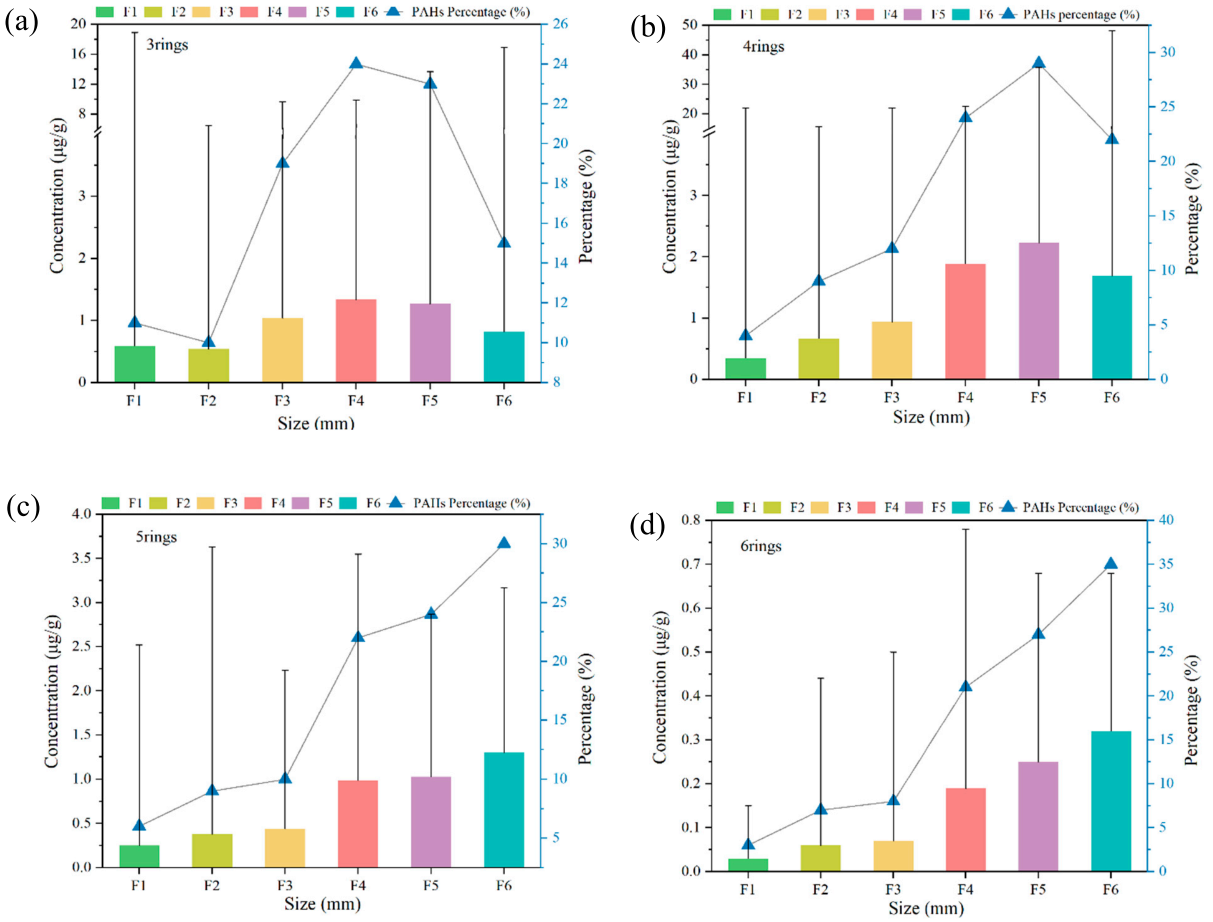

Figure S3. Distribution of PAHs in dust of different particle sizes across different ring numbers: (a) 3 rings; (b) 4 rings; (c) 5 rings; (d) 6 rings. The bars represent the concentration of PAHs ($\mu g \cdot g^{-1}$), and the dotted curves represent the percentage contribution of each ring-number group to the total $\sum_{17}$PAHs.

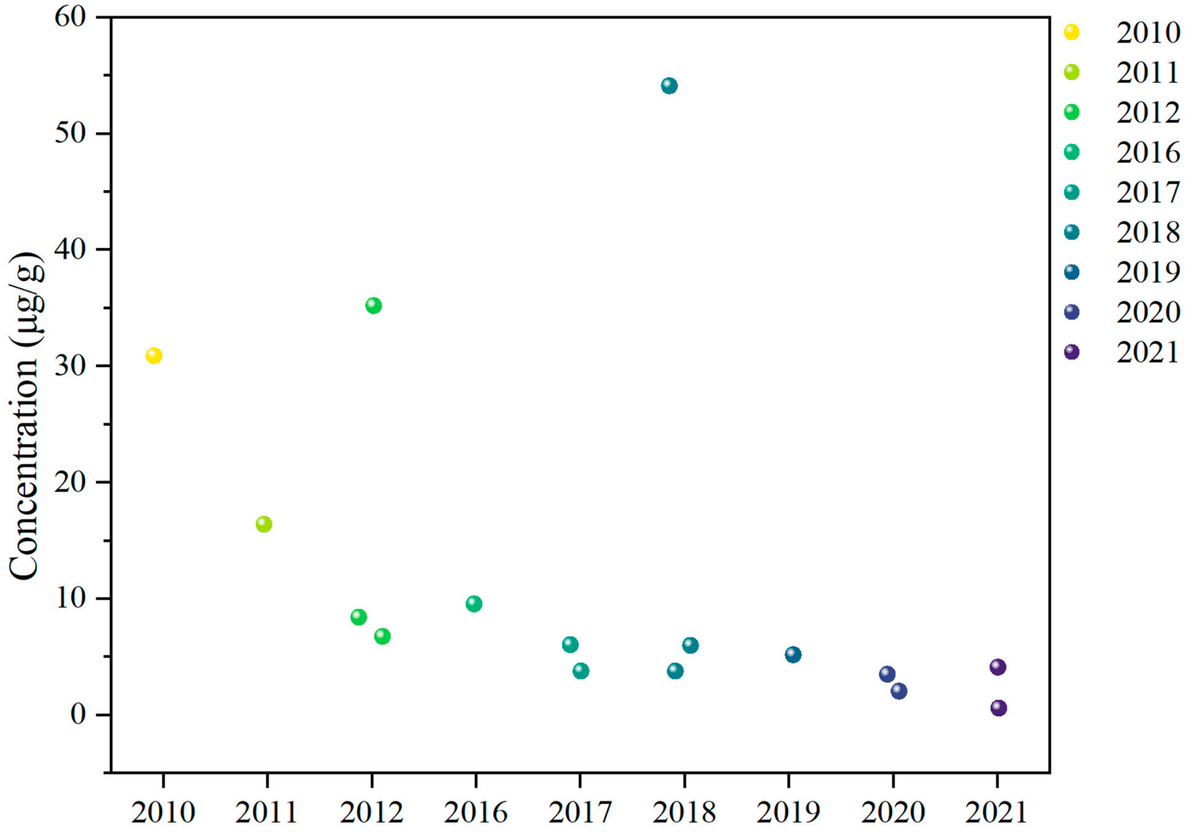

Figure S4. The total concentrations of PAHs in indoor dust from China during the period of 2010–2020. Detailed data can be found in Table S1.

S4. References

1. Qi, H.; Li, W.-L.; Zhu, N.-Z.; Ma, W.-L.; Liu, L.-Y.; Zhang, F.; Li, Y.-F. Concentrations and sources of polycyclic aromatic hydrocarbons in indoor dust in Chinal. *Sci. Total Environ.* **2014**, *491*, 100–107, doi:10.1016/j.scitotenv.2014.01.119.
2. Wang, B.L.; Pang, S.T.; Zhang, X.L.; Li, X.L.; Sun, Y.G.; Lu, X.M.; Zhang, Q.; Zhang, Z.D. Levels and neurodevelopmental effects of polycyclic aromatic hydrocarbons in settled house dust of urban dwellings on preschool–aged children in Nanjing, China. *Atmos. Pollut. Res.* **2014**, *5*, 292–302, doi:https://doi.org/10.5094/APR.2014.035.
3. Yang, Q.; Chen, H.; Li, B. Polycyclic Aromatic Hydrocarbons (PAHs) in Indoor Dusts of Guizhou, Southwest of China: Status, Sources and Potential Human Health Risk. *PLoS ONE* **2015**, *10*, doi:10.1371/journal.pone.0118141.
4. Yang, Z.-Z.; Li, Y.-F.; Fan, J. Polycyclic aromatic hydrocarbons in deposited bedroom dust collected from Xinxiang, a fast developing city in North China. *Environ. Monit. Assess.* **2015**, *187*, doi:10.1007/s10661-014-4150-6.
5. Wang, W.; Wu, F.; Zheng, J.; Wong, M.H. Risk assessments of PAHs and Hg exposure via settled house dust and street dust, linking with their correlations in human hair. *J. Hazard. Mater.* **2013**, *263*, 627–637, doi:10.1016/j.jhazmat.2013.10.023.
6. Wu, Y.; Hu, Q.; Zeng, X.; Xu, L.; Liang, Y.; Yu, Z. Co-occurrence of polycyclic aromatic hydrocarbons and their oxygenated derivatives in indoor dust from various microenvironments in Guangzhou, China: levels, sources, and potential human health risk. *Environ. Sci. Pollut. Res.* **2023**, *30*, 57006–57016, doi:10.1007/s11356-023-26476-6.
7. Huang, Q.; Zhao, T.; Qi, A.A.; Gao, H.L.; Zhang, W.; Duan, S.F.; Wang, P.C.; Wang, Y.M.; Zhang, X.F.; Wang, W.X.; et al. Comparison of indoor and outdoor polycyclic aromatic hydrocarbons from multiple urban residences in Northern China: Coastal versus inland area. *Build. Environ.* **2022**, *212*, doi:10.1016/j.buildenv.2022.108800.
8. Yang, Y.; Wang, Y.; Tan, F.; Zhang, Z.; Rodgers, T.F.M.; Chen, J. Pet hair as a potential sentinel of human exposure: Investigating partitioning and exposures from OPEs and PAHs in indoor dust, air, and pet hair from China. *Sci. Total Environ.* **2020**, *745*, doi:10.1016/j.scitotenv.2020.140934.
9. Wang, X.; Wang, X.; Qi, J.; Gong, S.; Wang, C.; Li, L.; Fan, L.; Liu, H.; Cao, Y.; Liu, M.; et al. Levels, distribution, sources and children health risk of PAHs in residential dust: A multi-city study in China br. *Sci. Total Environ.* **2023**, *862*, doi:10.1016/j.scitotenv.2022.160760.
10. Liu, Y.; Mao, Y.; Xu, J.; Chen, W.M.; Hu, T.P.; Xu, C.Y.; Liu, W.J.; Qu, C.K.; Chen, W.; Zhang, J.Q.; et al. Health Risks Associated with Polycyclic Aromatic Hydrocarbons (PAHs) in Dustfall Collected from Universities in Wuhan, China. *ATMOSPHERE* **2022**, *13*, doi:10.3390/atmos13101707.
11. Wu, Z.; Lyu, H.; Guo, Y.; Man, Q.; Niu, H.; Li, J.; Jing, X.; Ren, G.; Ma, X. Polycyclic aromatic hydrocarbons and polybrominated diphenyl ethers inside university campus: Indoor dust-bound pollution characteristics and health risks to university student. *Build. Environ.* **2022**, *221*, doi:10.1016/j.buildenv.2022.109312.
12. Liu, B.L.; Huang, F.; Yu, Y.; Dong, W.H. Polycyclic Aromatic Hydrocarbons (PAHs) in Indoor Dust Across China: Occurrence, Sources and Cancer Risk Assessment. *Arch. Environ. Contam. Toxicol.* **2021**, *81*, 482–491, doi:10.1007/s00244-021-00881-9.
13. Liu, B.; Yu, X.; Lv, L.; Dong, W.; Chen, L.; Wu, W.; Yu, Y. A nationwide survey of polycyclic aromatic hydrocarbons (PAHs) in household dust in China: spatial distribution, sources, and health risk assessment. *Environ. Geochem. Health* **2023**, *45*, 4979–4993, doi:10.1007/s10653-023-01563-2.
14. Zhang, Y.; Tong, Y.J.; Cheng, F.; Shi, J.W.; Huang, J.H.; Yu, M.Q.; You, J. Occurrence of emerging contaminants in pet hair and indoor air: integrative health risk assessment using multiple ToxCast endpoints. *Environ. Sci.: Processes Impacts* **2023**, *25*, 1839–1849, doi:10.1039/d3em00182b.
